# *In vitro* fertilization (IVF): production of distant hybrids with suitable genome combination

**DOI:** 10.1270/jsbbs.25086

**Published:** 2026-06-20

**Authors:** Takashi Okamoto

**Affiliations:** 1 Department of Biological Sciences, Tokyo Metropolitan University, Tokyo 192-0397, Japan

**Keywords:** gamete, zygote, hybridization, *in vitro* fertilization, wheat, rice, maize

## Abstract

Hybridization generates biodiversity and wide hybridization plays a pivotal role in enhancing and broadening the useful attributes of crops. Hybridization can be achieved through sexual or somatic hybridization between distant species; however, sexual hybridization between intergeneric or inter-subfamily combinations often results in unsuccessful fertilization/embryogenesis due to pre- and/or post-fertilization barriers. In addition, as cytoplasmic organelles are maternally transmitted to the zygote via sexual reproduction, cytoplasmic genome hybridization is rare. Although somatic hybridization offers distinct advantages over sexual hybridization, the isolation and culture of protoplasts are not feasible in most plant species, including vital crops. Recently, allopolyploid wheat–rice or wheat–maize hybrid zygotes were produced by *in vitro* fertilization (IVF) using isolated wheat, rice, and maize gametes, and these hybrid zygotes developed into wheat cytoplasmic hybrids (cybrids) with wheat–rice or wheat–maize hybrid mitochondria. These indicate that the IVF system with isolated gametes provides a solution to limitations of fertilization barriers and one-way cytoplasmic inheritance in sexual and somatic hybridizations, and that the present rice–wheat cybrid, ^Oryza^Wheat, and maize–wheat, ^Zea^Wheat, will provide a new horizon for the utilization of inter-subfamily genetic resources.

## Introduction

Hybridization is a key strategy in plant breeding, as this process can increase genetic variation through sexual or somatic crosses between distant species. Sexual hybridization is generally conducted through cross-pollination, and the resulting hybrid zygote, which possesses totipotency for embryogenesis, can transmit heterogeneous genetic material to progeny. However, sexual hybridization between intergeneric or inter-subfamily combinations often results in unsuccessful fertilization/embryogenesis due to pre- and/or post-fertilization barriers (Haig and Westoby 1991, Morgan *et al.* 2010). In addition, as cytoplasmic organelles are maternally transmitted to the zygote during sexual reproduction, cytoplasmic genome hybridization is rare.

Meanwhile, somatic hybridization offers distinct advantages compared to sexual hybridization. This process involves fusing somatic protoplasts from different species, resulting in hybrid somatic cells with nuclear and cytoplasmic genomes from the respective species. However, isolation and culture of protoplasts is not feasible in most plant species. In addition, in the majority of monocotyledonous plants, including vital crops such as rice, maize, and wheat, regeneration from protoplasts remains either unattainable or inefficient (Davey *et al.* 2005, Eeckhaut *et al.* 2013).

Recently, using *in vitro* fertilization (IVF) with isolated wheat and rice gametes, we produced allopolyploid wheat–rice hybrid zygotes that developed into wheat cytoplasmic hybrids (cybrids) with wheat–rice hybrid mitochondria (Fig. 1; Maryenti *et al.* 2021, 2024). In addition, cybrids with wheat–maize hybrid mitochondria were also recently created in our laboratories (Onda *et al.* 2025). These indicate that the IVF system with isolated gamete provides a solution to limitations of fertilization barriers and one-way cytoplasmic inheritance in sexual and somatic hybridizations, and serves as a bridge between sexual crossing and somatic hybridization. Thus, after an explanation of the IVF system for plant gametes, this review addresses the current status of hybrid production via IVF. Thereafter, possible new plant cytoplasmic breeding via the IVF system and future perspectives for cybrid research are discussed.

## *In vitro* fertilization with plant gametes

Fertilization is the key event in the life cycle of higher organisms. In angiosperms, the female gametophyte, also called the embryo sac or megagametophyte, develops in the ovule, and fertilization and subsequent events, such as embryogenesis and endosperm development, occur within the embryo sac, which is deeply embedded in the ovular tissue (Raghavan 2003, Russell 1992). Meanwhile, difficulties in directly researching the biology of the embedded female gamete, zygote, and early embryo have impeded investigations into the molecular mechanisms of fertilization and embryogenesis. Therefore, such studies have been conducted predominantly by analyzing *Arabidopsis* mutants or transformants, coupled with live imaging (reviewed in Berger 2011, Hamamura *et al.* 2012, Maruyama *et al.* 2015). Alternatively, direct analyses using isolated gametes or zygotes are possible because procedures for isolating viable gametes have been established, and IVF systems using isolated gametes can be used to observe and analyze fertilization and post-fertilization processes directly (reviewed in Wang *et al.* 2006).

The IVF system used for angiosperms involves a combination of three basic microtechniques: (i) the isolation and selection of male and female gametes, (ii) the fusion of pairs of gametes, and (iii) single-cell cultures (Fig. 2; Kranz 1999). Procedures have been established in a wide range of plant species for the isolation of viable gametes, including both monocotyledonous and dicotyledonous plants (reviewed in Kranz 1999 and in Okamoto 2011). As for gamete fusion, the fusion procedure can be performed electrically (Kranz and Lörz 1993, Uchiumi *et al.* 2007) or chemically via calcium (Faure *et al.* 1994, Khalequzzaman and Haq 2005, Kranz and Lörz 1994), polyethylene glycol (PEG) (Sun *et al.* 1995, Tian and Russell 1997), or bovine serum albumin (BSA) (Peng *et al.* 2005), as plasma membranes of isolated gametes are partly or largely exposed, and plasma membranes between the isolated gametes can attach. Investigations with calcium-based fusion of maize gametes have shown that calcium influx is triggered by gamete fusion and that cell wall formation, an event known as egg activation, is induced by the calcium influx (Antoine *et al.* 2001). Moreover, fusion behavior and gamete interaction have been traced using a PEG-mediated gamete fusion procedure (Sun *et al.* 2000). However, as presented in Table 1, zygotes produced by calcium-, PEG-, or BSA-fusion appeared to become arrested in development (Kranz and Lörz 1994, Peng *et al.* 2005, Sun *et al.* 2000, Tian and Russell 1997). Importantly, among these four different procedures, only zygotes produced by electro-fusion are known to divide and develop into embryo-like structures and plantlets.

A complete IVF system that involves maize gametes and electrical fusion was first developed by Kranz and Lörz (1993), and rice and wheat IVF systems were also established by Uchiumi *et al.* (2007) and Maryenti *et al.* (2019), respectively, to take advantage of the abundant resources stemming from rice and wheat research. These electrofusion-based IVF systems have been successfully utilized to observe and investigate post-fertilization events, such as karyogamy (Faure *et al.* 1993, Ohnishi *et al.* 2014), egg activation and zygotic development (Kranz *et al.* 1995, Nakajima *et al.* 2010, Sato *et al.* 2010, Sukawa and Okamoto 2018), paternal chromatin decondensation in zygote nuclei (Scholten *et al.* 2002), microtubular architecture in egg cells and zygotes (Hoshino *et al.* 2004), ROS and mitochondrial dynamics (Aini *et al.* 2022, Rattanawong *et al.* 2021), fertilization-induced/suppressed gene expression (Okamoto *et al.* 2005, Toda *et al.* 2025), the contribution of parental genomes to zygotic development (Deushi *et al.* 2021, Toda *et al.* 2016, 2018), epigenetic resetting in early embryos (Jahnke and Scholten 2009), and the synergistical function of parental genomes (Akter *et al.* 2025, Masuo *et al.* 2025, Rahman *et al.* 2019).

In addition to investigations of post-fertilization events, IVF systems can be applied to generate new genotypes and/or hybrids, as gametes isolated from different species, such as maize and wheat, can be fused to produce distantly related hybrid zygotes beyond the reproductive barrier. Additionally, the combination and number of gametes for electro-fusion can be set according to the species and ploidy of interest. The following three sections describe the recent progress in cytoplasmic hybrids between “rice and wheat” and “maize and wheat” across subfamilies.

## Overcoming the hybridization defect between wheat and rice, and production of the rice–wheat cytoplasmic hybrid, ^Oryza^Wheat

Based on the availability of rice and wheat IVF systems, rice–wheat hybrid zygotes were produced by fusing a rice egg cell (Re) and a wheat sperm cell (Ws). Although the ReWs zygotes underwent several rounds of cell division, their developmental progressions ceased at the embryo-like stage (Maryenti *et al.* 2021). Fluorescence *in situ* hybridization (FISH) analyses using probes for the rice centromere sequence and/or the wheat genome were employed to determine the chromosome composition of cells in ReWs hybrid embryo-like structures. Notably, rice centromeres were not observed or detectable at the periphery or outside the nuclei in these cells. In contrast, the wheat-specific probe signals were stable and uniformly detected in all hybrid nuclei. This suggests that rice chromosomes are eliminated during the early developmental stage of the ReWs hybrid zygotes, and that the selective rice chromosome elimination in ReWs embryos resulted in cells possessing the cytonuclear environment of the wheat nucleus and rice cytoplasm (Fig. 3A). This allogenic cytonuclear environment was presumed to be the reason that ReWs embryos arrested at globular-like embryo stage because dysfunctional interactions between a cytoplasmic genome from one species and a nuclear genome from another often result in organelle dysfunction and, consequently, hybrid breakdown (Burton *et al.* 2013, Johnson 2010, Nakamura 2023). Therefore, an additional wheat egg cell was further fused with a ReWs zygote to supply wheat cytoplasmic genome into the hybrid zygote and to establish a nuclear–cytoplasmic (NC) relationship between wheat nucleus and wheat cytoplasm, possibly rescuing the developmental defect of ReWs embryos triggered by dysfunction of the NC relationship between wheat nucleus and rice cytoplasm (Fig. 3B).

The allopolyploid rice egg (Re)–wheat sperm (Ws)–wheat egg (We) hybrid zygotes, produced through the fusion of an extra We cell with a ReWs, were successfully developed and regenerated into the possible hybrid plantlets (Fig. 3B). In addition to the gamete combination of ReWsWe hybrid zygotes, alternative allopolyploid hybrid zygotes, termed double zygotes (DZs), consisting of a Re, a rice sperm (Rs), a We, and a Ws, also bypassed the hybridization defect in ReWs hybrid zygotes (Fig. 3C). Possible hybrid plants derived from ReWsWe zygotes and DZ zygotes were morphologically equivalent to wheat plants and fertile, and viable seeds were successfully harvested (Fig. 3D). In addition, flow cytometric analyses and chromosome number counting for these plants resulted in equivalent values with wheat, suggesting that the regenerated ReWsWe and DZ plants are equivalent to wheat plants (Maryenti *et al.* 2021).

## Wheat-rice hybrid mitochondria genome in ^Oryza^Wheat and stable transmission of the hybrid genome across generations

To examine the genomic status of the possible wheat–rice hybrids, short-read sequencing of genome DNA isolated from seven possible wheat–rice hybrid plant lines was conducted, and the genomic origin of the sequence reads was estimated (Maryenti *et al.* 2024). The results indicated that possible hybrids possess partial regions derived from rice mitochondrial genome, as well as whole wheat nuclear and cytoplasmic genomes (Fig. 4A). Interestingly, the retention rate of rice mitochondrial DNA ranged from 11 to 47% depending on the hybrid line. Long-read sequencing of the genome DNA and subsequent *in silico* analyses suggested that each partial DNA region with >2000 bp of rice mitochondrial genome is inserted at the putative hotspot region of wheat mitochondrial DNA (Fig. 4B). The hybrid mitochondrial DNA were visualized by FISH analyses with specific fluorescent probes for rice and mitochondrial DNA to root cells of the wheat–rice hybrids (Fig. 4C). These genomic and cytogenetic results indicate that the rice–wheat hybrids are cytoplasmic hybrid wheat possessing rice mitochondrial genome DNA regions. Therefore, we termed the cytoplasmic hybrid as ^Oryza^Wheat.

The transmission status of the rice mitochondrial genome DNA was examined in F_2_ and F_3_ generations using genomic PCR with primer sets specific to each mitochondrial region. The results suggested that the rice mitochondrial genome in ^Oryza^wheat was maintained and transmitted to their progenies (F_2_ and F_3_), although the retention profiles of the F_2_ and F_3_^Oryza^wheat generations displayed a possible loss of the insertional regions.

## Production of maize–wheat cytoplasmic hybrid, ^Zea^Wheat

Wheat belongs to the subfamily Pooideae (C3 plant) and grows in temperate and subarctic zones (Erenstein *et al.* 2022a, Huang *et al.* 2022). In contrast, maize, a C4 Poaceae plant belonging to the subfamily Panicoideae, exhibits high photosynthetic efficiency and the ability for growing in tropical zones (Erenstein *et al.* 2022b). Despite the valuable genetic resources in maize offers for enhancing the physiological and agricultural traits of wheat, transferring genetic material between wheat and maize remains challenging due to the hybridization barrier posed by chromosome elimination. Therefore, wheat and maize IVF systems were used to produce maize–wheat hybrids according to the procedures for ^Oryza^Wheat production.

When a hybrid zygote was produced by fusing a maize egg cell (Me) with a wheat sperm cell (Ws) and cultured, the MeWs zygotes arrested at the early cell mass stage (Fig. 5A; Onda *et al.* 2025). The developmental arrest of the MeWs embryo will also be attributable to the elimination of the maize genome during MeWs zygote development, as the coexistence of the maize nuclear genome with that of wheat results in the elimination of the maize genome during the development of wheat–maize hybrid zygotes (Laurie and Bennett 1988, Mochida *et al.* 2004). Developmental arrest of MeWs embryos by possible dysfunction of the NC-relationship between wheat and maize is consistent with that between wheat and rice in rice egg-wheat sperm (ReWs) hybrid embryos.

The developmental arrest was overcome by the fusion of an extra wheat egg cell (We) to MeWs zygotes probably via coexistence of wheat cytoplasm (Fig. 5B). In addition to the gamete combination of MeWeWs hybrid zygotes, alternative allopolyploid hybrid zygotes, termed DZs, consisting of a Me, a maize sperm (Ms), a We, and a Ws cell, were also bypassed the hybridization defect in MeWs hybrid zygotes (Onda *et al.* 2025).

All MeWeWs and DZ regenerated plants were morphologically similar to wheat plants, and flow cytometric analyses of these plants indicated that their DNA content was equivalent to that of wheat. Of the 40 tested plants (21 MeWeWs and 19 DZ plants), 37 were fertile, and viable seeds were successfully harvested (Fig. 5C), whereas the remaining three plants were infertile with a dwarf phenotype.

## Genome composition and photosynthetic type of ^Zea^Wheat

Using short-read sequencing of genome DNA isolated from eight possible maize–wheat hybrid plant lines, we found that the possible hybrid plants possess the whole wheat nuclear and cytoplasmic genomes and rice mitochondrial genome (Fig. 6A); this led us to name the cytoplasmic hybrid ^Zea^Wheat. Interestingly, the coverage rate of the maize mitochondrial genome in MeWeWs and DZ plants ranged from 30% to 93% (average: 75%), and the coverage rate in the ^Zea^Wheat hybrid was much higher than that in ^Oryza^Wheat (11% to 47%, average: 27%). Moreover, the maize mitochondrial DNA regions were found to be stably transmitted across generations (Fig. 6B). The FISH analyses of ^Zea^Wheat root cells with specific probes for maize and wheat mitochondrial DNAs indicated that maize and wheat mitochondria coexist, and that fusion between the maize and wheat mitochondria and subsequent recombination between the maize and wheat mitochondrial DNA was found to occur in the cybrid cells (Onda *et al.* 2025).

As ^Zea^Wheat possessed mitochondrial genomes of both C_3_ plants (wheat) and C_4_ plants (maize), the A/Ci response was measured to estimate the photosynthetic type of the cybrid plants. Although the maize plant performed at a better photosynthetic rate than wheat at low CO_2_ concentrations, the ^Zea^Wheat hybrid showed photosynthetic rates similar to those of wheat at low CO_2_ concentrations (Fig. 6C), suggesting that ^Zea^Wheat performs C_3_-like photosynthesis even if the cybrids possess maize mitochondria. The δ^13^C was also analyzed using EA/IRMS to estimate the carbon fixation pathway in the wheat–maize cybrid plants. Maize and wheat showed a high δ^13^C (–10.3 to –9.2‰) and a low δ^13^C (–36.8 to –32.9‰), respectively, and ^Zea^Wheat lines showed a low δ^13^C ranging from –37.8 to –31.3‰, equivalent to that of wheat (Fig. 6D), suggesting that the cybrid plants fix CO_2_ via a C_3_-type pathway.

## Discussion and Future Perspectives

### Mitochondrial hybridization across subfamily and physiological/agronomical traits

Cytoplasmic organelles, mitochondria and plastids, are essential for energy production, nutrient sensing, metabolism, and stress responses (Liberatore *et al.* 2016). It has been demonstrated that the cytoplasm evolves adaptively to the environment, conferring survival and fitness advantages (Bock *et al.* 2014, Leinonen *et al.* 2011, Sambatti *et al.* 2008), and that wheat cytoplasm substitution lines, in which *Aegilops* cytoplasm replaces wheat cytoplasm, exhibit distinct phenotypes, such as cytoplasmic male sterility (CMS), delayed heading, and parthenogenesis (Tsunewaki 2009, Tsunewaki *et al.* 2002). However, studies on plasmotypic effects have largely been limited to closely related species capable of cross-pollination. Therefore, examining the impact of cytoplasmic hybridization between distantly related species from different subfamilies is of considerable interest.

Cytoplasmic hybridization increases the nucleus-cytoplasm (NC) relationship and combination, and the variations in the NC relationship/combination are relevant from both agricultural and evolutionary perspectives (Bock *et al.* 2014, Levings 1990, Nakamura 2023). Moreover, the impacts of plasmotypic variation are particularly pronounced in fluctuating and stressful environments, because plasmotypes serve as reservoirs of variation that are predominantly expressed under certain conditions (Flood *et al.* 2020). These suggest that distant cytoplasmic hybridization among major crops will be highly important for broadening the variety of NC combinations and the subsequent physiological and agricultural traits of crops. To investigate the effects of the hybrid mitochondrial genome hybridization on the physiological and agronomic characteristics of cybrid wheat, comprehensive phenotypic evaluations and stress tolerance trials of the F_2_ generation and beyond are currently underway in our laboratories. The cybrids display a wide range of phenotypic variation, with several lines outperforming control wheat in biomass accumulation, heading date, and salt resistance, suggesting that mitochondrial genome diversity generates phenotypic diversity (Ikpa-Agodo *et al.* 2025, Nowroz *et al.* 2025, Sugiura *et al.* 2025). Molecular machineries for hybrid mitochondria–nuclear interactions related to these characteristic traits will be elucidated in the near future.

### Wheat as an ideal plant material for increasing cytoplasmic variety via the IVF system

It has been demonstrated that hybridization between wheat and distantly related species, such as *Hordeum bulbosum*, rice, maize, pearl millet, sorghum, *Coix lacryma-jobi*, and *Imperata cylindrica*, eliminates the non-wheat genome (Barclay 1975, Inagaki and Mujeeb-Kazi 1995, Ishii *et al.* 2010, 2016, Komeda *et al.* 2007, Maryenti *et al.* 2024, Mochida and Tsujimoto 2001, Mochida *et al.* 2004). The stable wheat genome characteristics upon hybridization are extremely suitable for generating cybrids that incorporate the mitochondrial genome of other species while retaining a wheat nuclear background. In addition to rice and maize, egg cells can be isolated from other Poaceae species, such as pearl millet, sugarcane, barley, sorghum, and setaria (Fig. 7), and these egg cells can also be utilized to create new cytoplasmic hybrid wheat.

Importantly, hybrid plants produced by *in vitro* fusion of gametes from plant species within the same Poaceae family are free from legislative concerns related to genetically modified organisms (Jones 2015). This creates the possibility of cultivating these hybrid plants in open fields immediately after their production.

### Research toward the elucidation of the function of the hybrid mitochondria in wheat cybrids

In addition to creating mitochondrial genome diversity using the IVF system with gametes from different species, the genome-editing technique can be utilized as a powerful tool to enhance or suppress the function of allogenic mitochondrial genomes. Indeed, we have developed a PEG–Ca^2+^-mediated transfection system to deliver macromolecules, including DNA, RNA, and proteins, into rice and maize zygotes produced *in vitro* and isolated from pollinated ovaries, respectively (Koiso *et al.* 2017). Importantly, genome editing of rice and maize has also been achieved via the delivery of a plasmid DNA vector with a CRISPR/Cas9-guideRNA expression cassette or CRISPR/Cas9-guideRNA ribonucleoproteins into rice zygotes (Toda *et al.* 2019) and maize zygotes (Yamada *et al.* 2024). However, the CRISPR/Cas9 system remains only adaptable to the nuclear genome. Therefore, an alternative nuclease, transcription activator-like effector nuclease tagged with the mitochondrial targeting sequence (mitoTALEN), has been used to edit mitochondrial DNA in plants and animals (reviewed in Arimura and Nakazato 2024). In angiosperms, open reading frame 79 (orf79) on the rice mitochondrial DNA was first successfully edited by Kazama *et al.* (2019). Delivering a DNA vector containing a mitoTALEN expression cassette into hybrid zygotes is a possible approach to edit hybrid mitochondrial DNA in cybrids toward elucidation of the function of hybrid mitochondria in cybrid wheat.

### Application of IVF technology for production of doubled haploid and for regeneration of recalcitrant genotypes/cultivars

Production of haploid and subsequent genome doubling, resulting in emergence of doubled haploid, is an efficient approach for instant fixation of genome composition. Therefore, doubled haploid technology enabling efficient development of pure lines has been considered as an important procedure for crop breeding (Qu *et al.* 2024, Seguí-Simarro *et al.* 2021). Recently, we reported an alternative utilization of the IVF technology (egg cell isolation and subsequent egg cell culture) for production of haploid and doubled haploid rice plants. Upon cold treatment, egg cells isolated from japonica (cv. Nipponbare) and indica (cv. Kasalath) subspecies of rice (*Oryza sativa*) autonomously divided, developed and regenerated into doubled haploid or haploid plant with or without genome duplication, respectively, during egg cell culture (Rattanawong *et al.* 2026). These suggest that environmental stimuli such as cold stress, cell isolation and/or culture convert cell fate of egg cell from quiescence to proliferative state, and that direct production of doubled haploid form egg cell will become a powerful technology for haploid breeding. Although report for induction of autonomous development (parthenogenetic potential) of isolated egg cells has been still limited to rice, egg cells isolated from other crops can be subjected to abiotic stress treatment and subsequent culture to examine their parthenogenetic potentials.

Although cell/tissue culture and its subsequent regeneration into plantlets are mostly required for producing transgenic and/or genome-edited crops, these procedures are major technical hurdle for recalcitrant genotypes/cultivars. IVF technology may be useful for obtaining regenerated plantlets form recalcitrant genotype/cultivars, as genome edited maize plants were successfully obtained by culture of recalcitrant B73 maize zygotes, into which Cas9-gRNA ribonucleoproteins were delivered (Yamada *et al.* 2024).

## Author Contribution Statement

T.O. wrote the manuscript.

## Figures and Tables

**Fig. 1.**
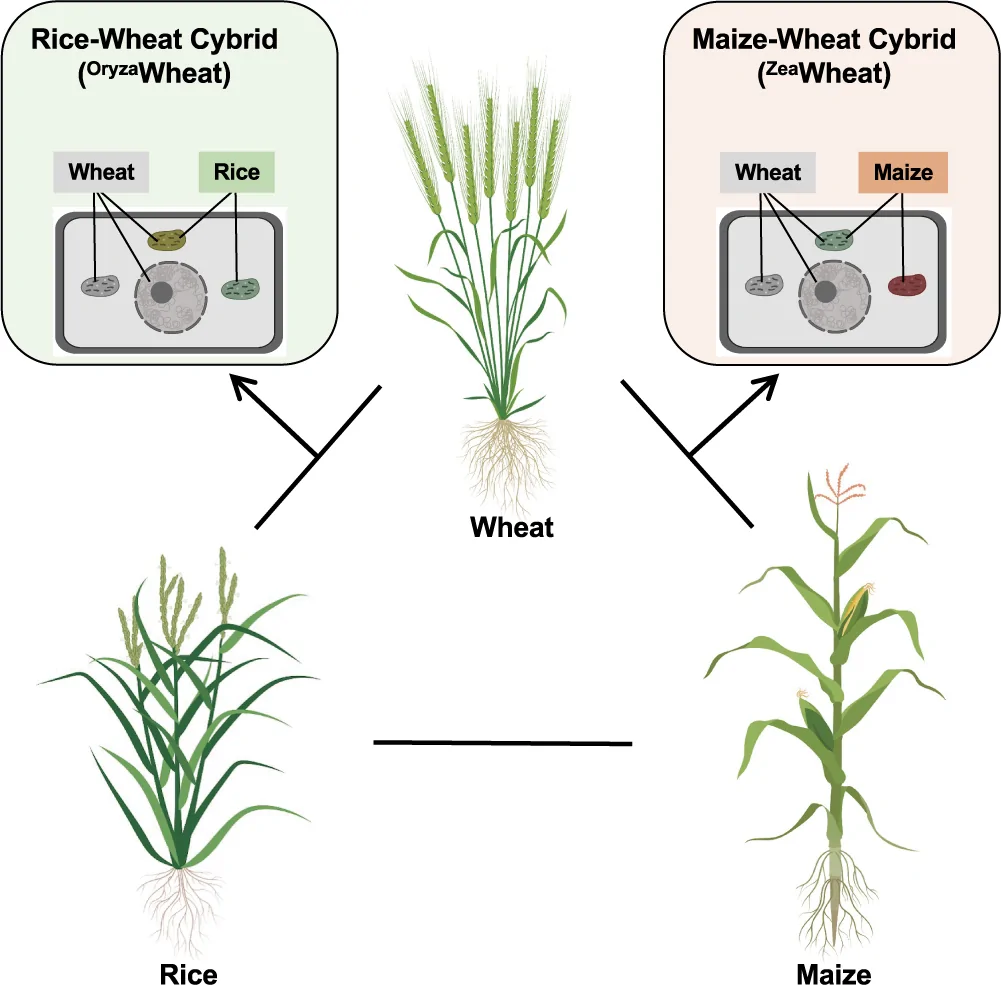
Cytoplasmic hybrids, ^Oryza^Wheat and ^Zea^Wheat, across three major crops belonging to different subfamilies.

**Fig. 2.**
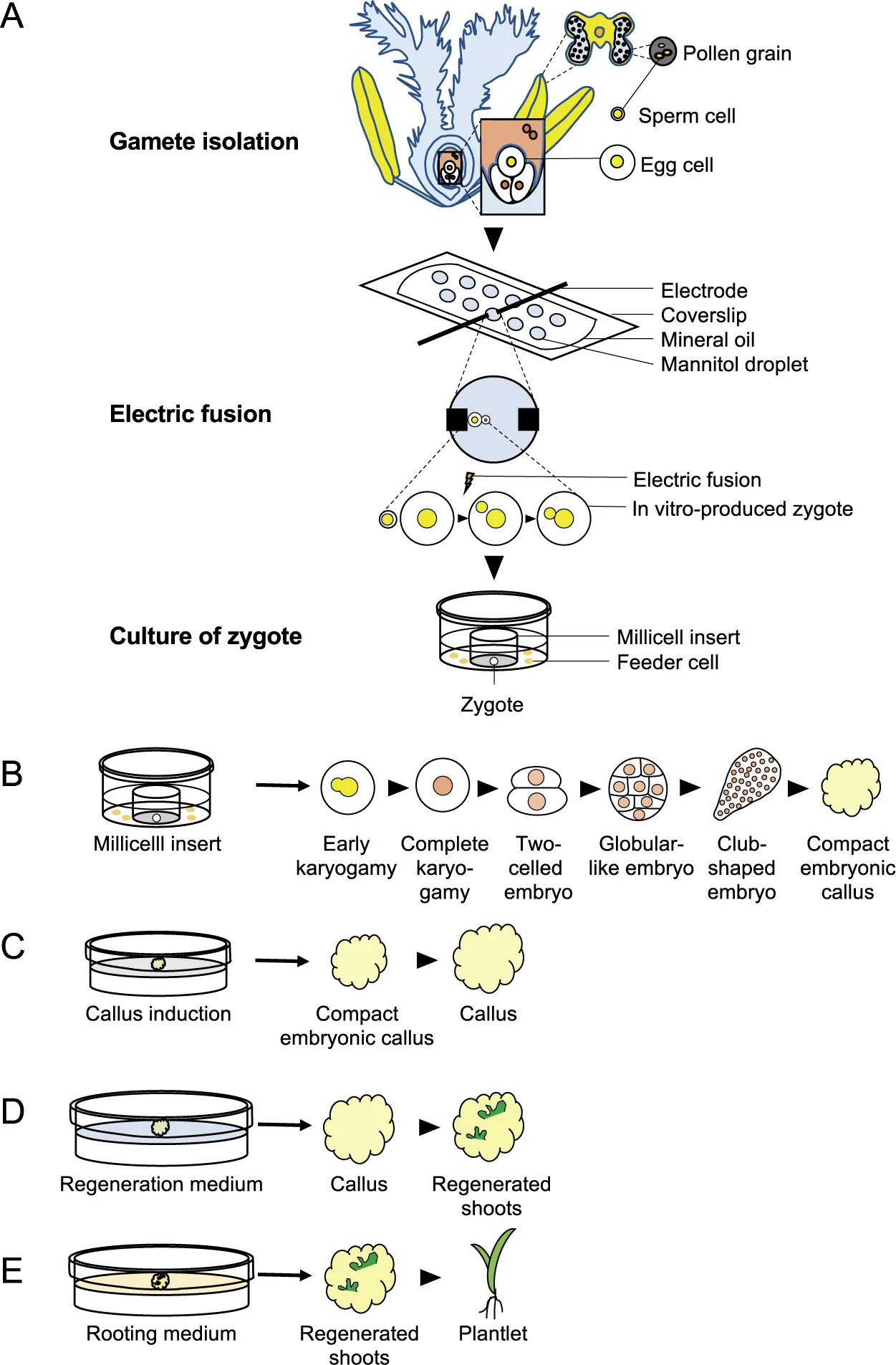
Schematic illustration of procedures for an *in vitro* fertilization system with wheat gametes. (A) Gametes, an egg cell and a sperm cell, are isolated from an unpollinated flower and transferred into a droplet of mannitol solution overlaid with mineral oil on a coverslip. The gametes are positioned in the center of a droplet, fixed at one of the electrodes under an alternating current field, and fused by multiple direct current pulses. The zygote produced *in* *vitro* was cultured on the membrane of a Millicell insert containing N6Z medium and feeder cells. (B) A zygote divides into a compact embryonic callus through early embryonic development in the Millicell insert. (C) A compact embryonic callus develops on the callus induction medium. (D, E) Thereafter, the callus is transferred onto regeneration and rooting medium to obtain regenerated shoots and plantlets, respectively.

**Fig. 3.**
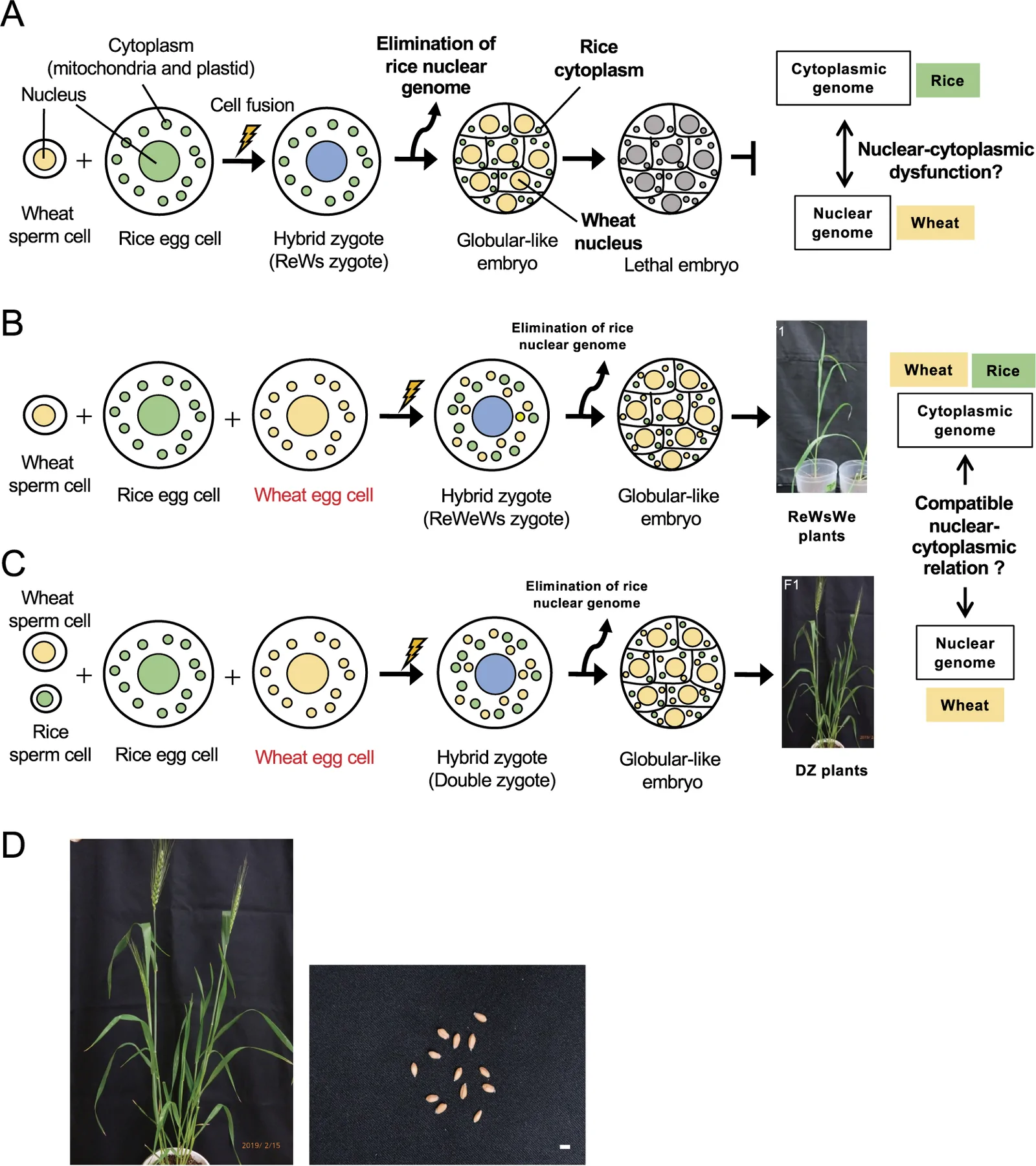
Schematic representation of the effects of gamete combination on the developmental profiles of wheat–rice hybrid zygotes (A–C) and mature plants regenerated from the hybrid zygotes and harvested seeds (D). (A) Hybrid zygotes derived from one rice egg (Re) and one wheat sperm (Ws) cease their development at the globular embryo-like structure stage after a selective elimination of rice chromosomes. (B) Hybrid incompatibility of the ReWs zygote presented in panel (A) can be overcome by additional fusion of one wheat egg cell (We) to the wheat–rice hybrid zygote. (C) Fusion of wheat and rice gamete sets, We, Ws cell, Re cell, and rice sperm cell (Rs), results in fusion products between wheat and rice zygote. The wheat–rice double zygote (DZ) also develops and regenerates into a mature plant via possible elimination of rice chromosomes. (D) Representative image of the regenerated plants and harvested seeds. Scale bar in D, 5 mm.

**Fig. 4.**
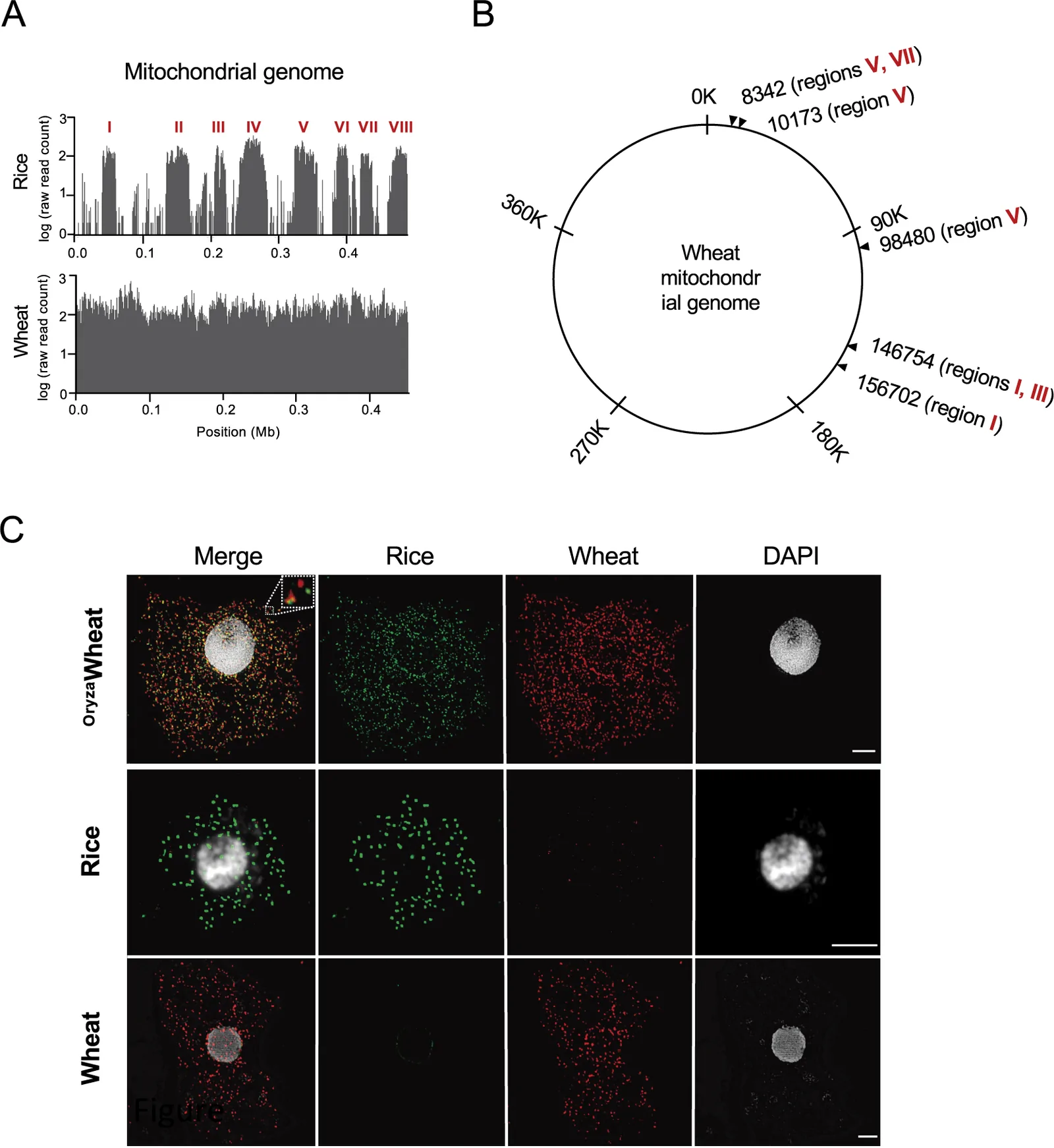
Genome composition of the rice–wheat hybrid. (A) Mapping pattern of the short-read sequences derived from the genome DNA of a possible rice–wheat hybrid onto rice (upper panel) and wheat (bottom panel) mitochondrial genomes. Eight DNA regions in the rice mitochondrial genome are numbered as regions I–VIII. (B) Putative insertion sites in the rice mitochondrial DNA regions in the wheat mitochondrial genome. Five putative hotspot sites where the rice mitochondrial genome tends to be inserted into the wheat mitochondrial genome are indicated by the black triangles, and the putatively inserted DNA regions from the rice mitochondrial genome are shown in parentheses after each possible hotspot. (C) Fluorescence *in situ* hybridization (FISH) analyses for rice and wheat mitochondrial genomes in cells of hybrid plants. FISH analyses using specific fluorescent probes for rice and wheat mitochondrial genomic DNA were conducted on root cells of rice–wheat hybrid plants (^Oryza^Wheat), rice plants, and wheat plants. Scale bars, 10 μm.

**Fig. 5.**
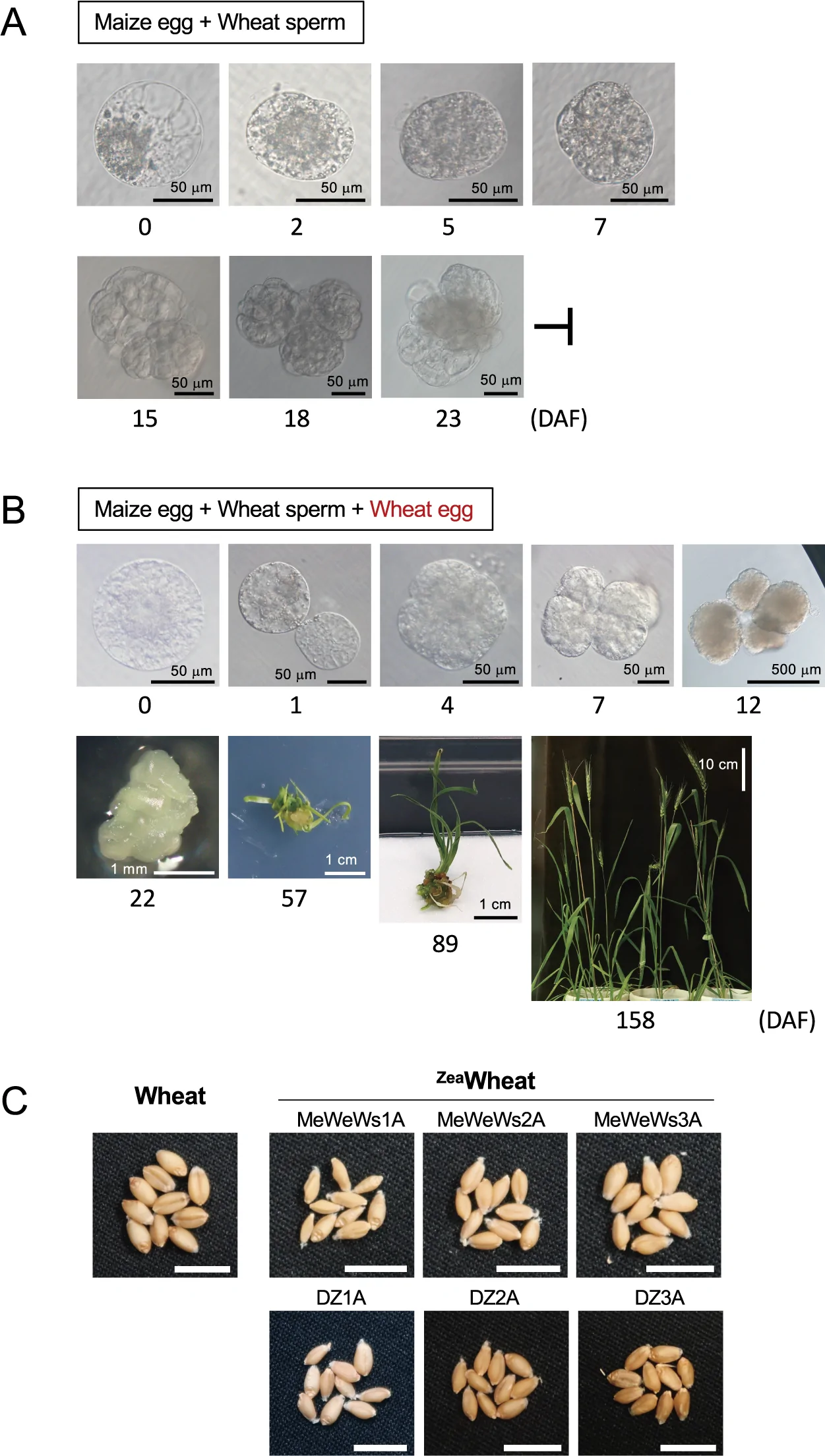
Developmental profiles of wheat–maize hybrid zygotes (A, B) and harvested seeds (C). (A) MeWs zygotes developed into globular-like embryos and early cell masses. Thereafter, the early cell masses became highly vacuolated and arrested. (B) MeWeWs zygotes developed into globular-like embryos, early cell masses, cell masses, compact calli, and calli. After regenerated shoots and the roots were formed from the calli, the regenerated plantlets grew into mature plants with fertile flowers. (C) Harvested seeds from six independent plants derived from hybrid zygotes. DAF: day after fusion. Scale bars in C, 1 cm.

**Fig. 6.**
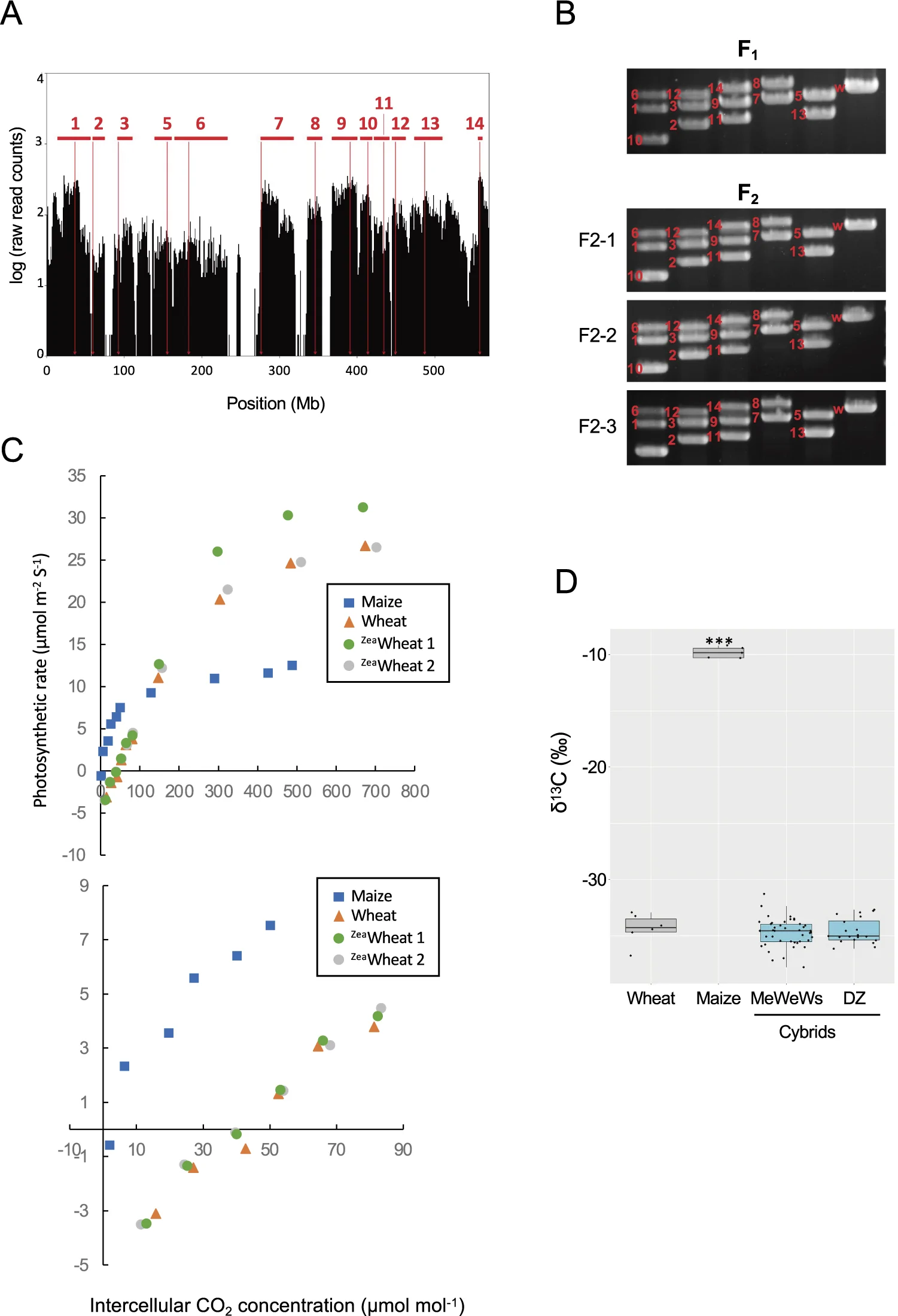
Mitochondrial genome composition of wheat–maize hybrid plants (A), transmission of maize mtDNA (B), and photosynthetic rate and stable carbon isotope ratios in wheat–maize cybrid plants (C, D). (A) Mapping profiles of sequence reads from the genomic DNA of a hybrid plant onto the maize mitochondrial genome. The positions of the PCR primer sets used in panel B were indicated by red-colored numbers. (B) Genomic PCR using gDNA from hybrid plants at F_1_ and F_2_ generations. (C) Response of photosynthetic rate to intercellular CO_2_ concentration (Ci) at 23°C and 1800 μmol m^–2^ s^–1^ light intensity for a wild-type maize (C_4_), wild-type wheat (C_3_), and two F_3_ cybrids. The low CO_2_ portion of the A versus Ci response is shown in the bottom panel. (D) Stable carbon-isotope ratios of wheat–maize F_1_ cybrid plants. Dunnett’s test, *p < 0.05, **p < 0.01, ***p < 0.001 vs. Fielder. wheat: n = 6, maize: n = 5, MeWeWs: n = 42, DZ: n = 20.

**Fig. 7.**
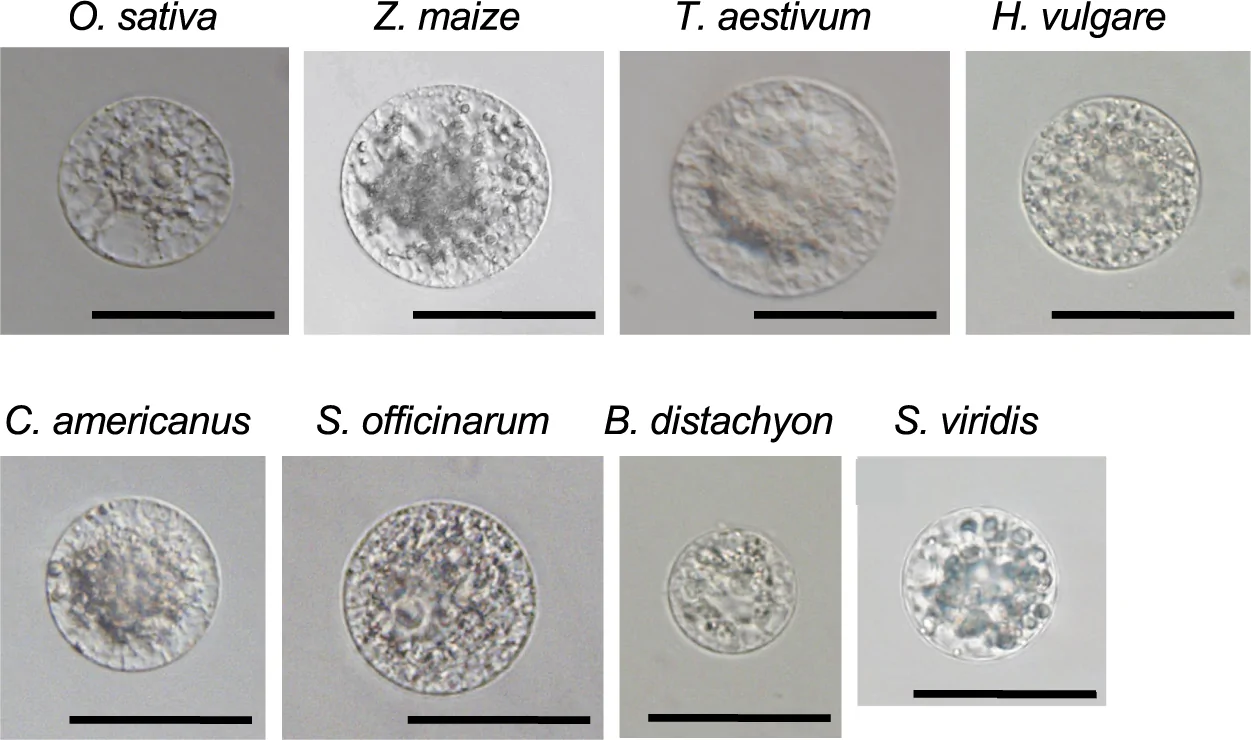
Egg cells isolated from unpollinated flowers of rice (*Oryza sativa* L.), maize (*Zea mays* L.), wheat (*Triticum aestivum* L.), barley (*Hordeum vulgare*), pearl millet (*Cenchrus americanus*), sugarcane (*Saccharum officinarum*), *Brachypodium distachyon*, and *Setaria viridis*. Scale bars, 50 μm.

**Table 1.** Procedures of *in vitro* fusion of isolated gametes and the developmental potential of the produced zygotes

Fusion procedure	Species	Developmental progression of fused gametes (zygotes)					References
		Gamete fusion	Karyogamy	Two-celled embryo	Cell mass	Plantlet	
Calcium-fusion	Maize	○	○	—	—	—	Kranz and Lörz 1994
	Maize	○	—	—	—	—	Faure *et al.* 1994
	Rice	○	—	—	—	—	Khalequzzaman and Haq 2005
PEG-fusion	Maize	○	—	—	—	—	Sun *et al.* 1995
BSA-fusion	Maize	○	—	—	—	—	Peng *et al.* 2005
Electro-fusion	Maize	○	○	○	○	○	Kranz and Lörz 1993
	Rice	○	○	○	○	○	Uchiumi *et al.* 2007
	Wheat	○	○	○	○	○	Maryenti *et al.* 2019

Circles indicates the developmental stages into which zygotes produced by respective fusion procedures develop. Dashes means the developmental stages into which zygotes produced by respective fusion procedures cannot develop.

## References

[B1] Aini, H., Y. Sato, K. Uno, T. Higashiyama and T. Okamoto (2022) Dynamics of mitochondrial distribution during development and asymmetric division of rice zygotes. Plant Reprod 35: 47–60. 10.1007/s00497-021-00430-3 34633536

[B2] Akter, N., T. Tezuka, K. Rattanawong, A. Satoh, A. Kinoshita, Y. Sato and T. Okamoto (2025) Role of paternal *Oryza sativa Baby Booms* (*OsBBMs*) in initiating *de novo* gene expression and regulating early zygotic development in rice. Plant J 122: e70305. 10.1111/tpj.70305 PMC12201997 40570546

[B3] Antoine, A.F., J.E. Faure, C. Dumas and J.A. Feijo (2001) Differential contribution of cytoplasmic Ca^2+^ and Ca^2+^ influx to gamete fusion and egg activation in maize. Nat Cell Biol 3: 1120–1123. 10.1038/ncb1201-1120 11781574

[B4] Arimura, S. and I. Nakazato (2024) Genome editing of plant mitochondrial and chloroplast genomes. Plant Cell Physiol 65: 477–483. 10.1093/pcp/pcad162 PMC11094758 38113380

[B5] Barclay, I.R. (1975) High frequencies of haploid production in wheat (*Triticum aestivum*) by chromosome elimination. Nature 256: 410–411.

[B6] Berger, F. (2011) Imaging fertilization in flowering plants, not so abominable after all. J Exp Bot 62: 1651–1658. 10.1093/jxb/erq305 20952626

[B7] Bock, D.G., R.L. Andrew and L.H. Rieseberg (2014) On the adaptive value of cytoplasmic genomes in plants. Mol Ecol 23: 4899–4911. 10.1111/mec.12920 25223488

[B8] Burton, R.S., R.J. Pereira and F.S. Barreto (2013) Cytonuclear genomic interactions and hybrid breakdown. Annu Rev Ecol Evol Syst 44: 281–302.

[B9] Davey, M.R., P. Anthony, J.B. Power and K.C. Lowe (2005) Plant protoplasts: status and biotechnological perspectives. Biotechnol Adv 23: 131–171. 10.1016/j.biotechadv.2004.09.008 15694124

[B10] Deushi, R., E. Toda, S. Koshimizu, K. Yano and T. Okamoto (2021) Effect of paternal genome excess on the developmental and gene expression profiles of polyspermic zygotes in rice. Plants 10: 255. 10.3390/plants10020255 PMC7911625 33525652

[B11] Eeckhaut, T., P.S. Lakshmanan, D. Deryckere, E. Van Bockstaele and J. Van Huylenbroeck (2013) Progress in plant protoplast research. Planta 238: 991–1003. 10.1007/s00425-013-1936-7 23955146

[B12] Erenstein, O., M. Jaleta, K.A. Mottaleb, K. Sonder, J. Donovan and H.J. Braun (2022a) Global trends in wheat production, consumption and trade. *In*: Reynolds, M.P. and H.J. Braun (eds.) Wheat Improvement, Springer, Cham, Switzerland, pp. 47–66.

[B13] Erenstein, O., M. Jaleta, K. Sonder, K. Mottaleb and B.M. Prasanna (2022b) Global maize production, consumption and trade: trends and R&D implications. Food Secur 14: 1295–1319.

[B14] Faure, J.E., H.L. Mogensen, C. Dumas, H. Lörz and E. Kranz (1993) Karyogamy after electrofusion of single egg and sperm cell protoplasts from maize: cytological evidence and time course. Plant Cell 5: 747–755. 10.1105/tpc.5.7.747 PMC160313 12271085

[B15] Faure, J.E., C. Digonnet and C. Dumas (1994) An in vitro system for adhesion and fusion of maize gametes. Science 263: 1598–1600. 10.1126/science.263.5153.1598 17744790

[B16] Flood, P.J., T.P.J.M. Theeuwen, K. Schneeberger, P. Keizer, W. Kruijer, E. Severing, E. Kouklas, J.A. Hageman, R. Wijfjes, V. Calvo-Baltanas et al. (2020) Reciprocal cybrids reveal how organellar genomes affect plant phenotypes. Nat Plants 6: 13–21. 10.1038/s41477-019-0575-9 31932677

[B17] Haig, D. and M. Westoby (1991) Genomic imprinting in endosperm: its effect on seed development in crosses between species, and between different ploidies of the same species, and its implications for the evolution of apomixis. Philos Trans R Soc Lond B Biol Sci 333: 1–13.

[B18] Hamamura, Y., S. Nagahara and T. Higashiyama (2012) Double fertilization on the move. Curr Opin Plant Biol 15: 70–77. 10.1016/j.pbi.2011.11.001 22153653

[B19] Hoshino, Y., S. Scholten, P. von Wiegen, H. Lörz and E. Kranz (2004) Fertilization-induced changes in the microtubular architecture of the maize egg cell and zygote—an immunocytochemical approach adapted to single cells. Sex Plant Reprod 17: 89–95.

[B20] Huang, W., L. Zhang, J.T. Columbus, Y, Hu, Y. Zhao, L. Tang, Z. Guo, W. Chen, M. McKain, M. Bartlett et al. (2022) A well-supported nuclear phylogeny of Poaceae and implications for the evolution of C_4_ photosynthesis. Mol Plant 15: 755–777. 10.1016/j.molp.2022.01.015 35093593

[B21] Ikpa-Agodo, K., R. Sugiura, N. Onda, A. Satoh, T. Maryenti, N. Mohamed, T. Okamoto and T. Ishii (2025) Phenotypic and metabolic effect of hybrid mitochondria of wheat and maize or pearl millet in *in vitro* fertilization-derived wheat cybrids. Breed Res 27 (Suppl 2): 78.

[B22] Inagaki, M. and A. Mujeeb-Kazi (1995) Comparison of polyhaploid production frequencies in crosses of hexaploid wheat with maize, pearl millet and sorghum. Japan J Breed 45: 157–161.

[B23] Ishii, T., T. Ueda, H. Tanaka and H. Tsujimoto (2010) Chromosome elimination by wide hybridization between Triticeae or oat plant and pearl millet: pearl millet chromosome dynamics in hybrid embryo cells. Chromosome Res 18: 821–831. 10.1007/s10577-010-9158-3 20953694

[B24] Ishii, T., R. Karimi-Ashtiyani and A. Houben (2016) Haploidization via chromosome elimination: Means and mechanisms. Ann Rev Plant Biol 67: 421–438. 10.1146/annurev-arplant-043014-114714 26772657

[B25] Jahnke, S. and S. Scholten (2009) Epigenetic resetting of a gene imprinted in plant embryos. Curr Biol 19: 1677–1681. 10.1016/j.cub.2009.08.053 19781944

[B26] Johnson, N.A. (2010) Hybrid incompatibility genes: Remnants of a genomic battlefield? Trends Genet 26: 317–325. 10.1016/j.tig.2010.04.005 20621759

[B27] Jones, H.D. (2015) Regulatory uncertainty over genome editing. Nat Plants 1: 14011. 10.1038/nplants.2014.11 27246057

[B28] Kazama, T., M. Okuno, Y. Watari, S. Yanase, C. Koizuka, Y. Tsuruta, H. Sugaya, A. Toyoda, T. Itoh, N. Tsutsumi et al. (2019) Curing cytoplasmic male sterility via TALEN-mediated mitochondrial genome editing. Nat Plants 5: 722–730. 10.1038/s41477-019-0459-z 31285556

[B29] Khalequzzaman, M. and N. Haq (2005) Isolation and in vitro fusion of egg and sperm cells in *Oryza sativa*. Plant Physiol Biochem 43: 69–75. 10.1016/j.plaphy.2005.01.001 15763668

[B30] Koiso, N., E. Toda, M. Ichikawa, N. Kato and T. Okamoto (2017) Development of gene expression system in egg cells and zygotes isolated from rice and maize. Plant Direct 1: e00010. 10.1002/pld3.10 PMC6508540 31245659

[B31] Komeda, N., H.K. Chaudhary, G. Suzuki and Y. Mukai (2007) Cytological evidence for chromosome elimination in wheat × *Imperata cylindrica* hybrids. Genes Genet Syst 82: 241–248. 10.1266/ggs.82.241 17660694

[B32] Kranz, E. (1999) In vitro fertilization with isolated single gametes. Methods Mol Biol 111: 259–267. 10.1385/1-59259-583-9:259 10080993

[B33] Kranz, E. and H. Lörz (1993) In vitro fertilization with isolated, single gametes results in zygotic embryogenesis and fertile maize plants. Plant Cell 5: 739–746. 10.1105/tpc.5.7.739 PMC160312 12271084

[B34] Kranz, E. and H. Lörz (1994) *In vitro* fertilization of maize by single egg and sperm cell protoplast fusion mediated by high calcium and high pH. Zygote 2: 125–128. 10.1017/s0967199400001878 7874454

[B35] Kranz, E., P. von Wiegen and H. Lörz (1995) Early cytological events after induction of cell division in egg cells and zygote development following *in vitro* fertilization with angiosperm gametes. Plant J 8: 9–23.

[B36] Laurie, D.A. and M.D. Bennett (1988) The production of haploid wheat plants from wheat × maize crosses. Theor Appl Genet 76: 393–397. 10.1007/BF00265339 24232203

[B37] Leinonen, P.H., D.L. Remington and O. Savolainen (2011) Local adaptation, phenotypic differentiation, and hybrid fitness in diverged natural populations of *Arabidopsis lyrata*. Evolution 65: 90–107. 10.1111/j.1558-5646.2010.01119.x 20812972

[B38] Levings, C.S. (1990) The Texas cytoplasm of maize: cytoplasmic male sterility and disease susceptibility. Science 250: 942–947. 10.1126/science.250.4983.942 17746917

[B39] Liberatore, K.J., S. Dukowic-Schulze, M.E. Miller, C. Chen and S.F. Kianian (2016) The role of mitochondria in plant development and stress tolerance. Free Radic Biol Med 100: 238–256. 10.1016/j.freeradbiomed.2016.03.033 27036362

[B40] Maruyama, D., R. Völz, H. Takeuchi, T. Mori, T. Igawa, D. Kurihara, T. Kawashima, M. Ueda, M. Ito, M. Umeda et al. (2015) Rapid elimination of the persistent synergid through a cell fusion mechanism. Cell 161: 907–918. 10.1016/j.cell.2015.03.018 25913191

[B41] Maryenti, T., N. Kato, M. Ichikawa and T. Okamoto (2019) Establishment of an *in vitro* fertilization system in wheat (*Triticum aestivum* L.). Plant Cell Physiol 60: 835–843. 10.1093/pcp/pcy250 30605551

[B42] Maryenti, T., T. Ishii and T. Okamoto (2021) Development and regeneration of wheat–rice hybrid zygotes produced by *in vitro* fertilization system. New Phytol 232: 2369–2383. 10.1111/nph.17747 PMC9293317 34545570

[B43] Maryenti, T., S. Koshimizu, N. Onda, T. Ishii, K. Yano and T. Okamoto (2024) Wheat cybrid plants, ^Oryza^Wheat, regenerated from wheat–rice hybrid zygotes via in vitro fertilization system possess wheat–rice hybrid mitochondria. Plant Cell Physiol 65: 1344–1357. 10.1093/pcp/pcae074 PMC11369819 39107984

[B44] Masuo, S., N. Akter, S. Koshimizu, K. Rattanawong, T. Kitta, Y. Taki, A. Kinoshita, A. Satoh, H. Takasaki, M. Ikeda et al. (2025) *Oryza sativa* Baby Boom1 converts the developmental fate of rice egg cells from gametic into somatic status. iScience 28: 113621. 10.1016/j.isci.2025.113621 PMC12547165 41142992

[B45] Mochida, K. and H. Tsujimoto (2001) Production of wheat doubled haploids by pollination with Job’s tears (*Coix lachryma-jobi* L.). J Hered 92: 81–83. 10.1093/jhered/92.1.81 11336235

[B46] Mochida, K., H. Tsujimoto and T. Sasakuma (2004) Confocal analysis of chromosome behavior in wheat × maize zygotes. Genome 47: 199–205. 10.1139/g03-123 15060616

[B47] Morgan, E.R., G.M. Timmerman-Vaughan, A.J. Conner, W.B. Griffin and R. Pickering (2010) Plant interspecific hybridization: outcomes and issues at the intersection species. *In*: Plant Breeding Reviews, John Wiley & Sons, Inc., New York, pp. 161–220.

[B48] Nakajima, K., T. Uchiumi and T. Okamoto (2010) Positional relationship between the gamete fusion site and the first division plane in the rice zygote. J Exp Bot 61: 3101–3105. 10.1093/jxb/erq131 PMC2892148 20462944

[B49] Nakamura, C. (2023) Nucleus-cytoplasm compatibility: A genetic system underlying allopolyploid speciation as exemplified in alloplasmic lines of wheat. Cytologia 88: 189–195.

[B50] Nowroz, F., N. Onda, A. Satoh, T. Ishii and T. Okamoto (2025) Identification of salt tolerant maize-wheat (^Zea^Wheat) and pearl millet-wheat (^Cenchrus^Wheat) cybrid lines produced through in vitro fertilization system. Breed Res 27 (Suppl 2): 119.

[B51] Ohnishi, Y., R. Hoshino and T. Okamoto (2014) Dynamics of male and female chromatin during karyogamy in rice zygotes. Plant Physiol 165: 1533–1543. 10.1104/pp.114.236059 PMC4119036 24948834

[B52] Okamoto, T. (2011) In vitro fertilization with isolated rice gametes: production of zygotes and zygote and embryo culture. Methods Mol Biol 710: 17–27. 10.1007/978-1-61737-988-8_2 21207258

[B53] Okamoto, T., S. Scholten, H. Lörz and E. Kranz (2005) Identification of genes that are up- or down-regulated in the apical or basal cell of maize two-celled embryos and monitoring their expression during zygote development by a cell manipulation- and PCR-based approach. Plant Cell Physiol 46: 332–338. 10.1093/pcp/pci032 15695436

[B54] Onda, N., A. Satoh, F. Nowroz, T. Maryenti, R. Mega, T. Ishii and T. Okamoto (2025) Inter-subfamily cybrid plants between wheat and maize, ^Zea^wheat: production via an *in vitro* fertilization system, genome composition, and photosynthetic type. J Exp Bot 76: 5912–5929. 10.1093/jxb/eraf354 PMC12621097 40831004

[B55] Peng, X.B., M.X. Sun and H.Y. Yang (2005) A novel *in vitro* system for gamete fusion in maize. Cell Res 15: 734–738. 10.1038/sj.cr.7290343 16212880

[B56] Qu, Y., A.R. Fernie, J. Liu and J. Yan (2024) Doubled haploid technology and synthetic apomixis: Recent advances and applications in future crop breeding. Mol Plant 17: 1005–1018. 10.1016/j.molp.2024.06.005 38877700

[B57] Raghavan, V. (2003) Some reflections on double fertilization, from its discovery to the present. New Phytol 159: 565–583. 10.1046/j.1469-8137.2003.00846.x 33873607

[B58] Rahman, M.H., E. Toda, M. Kobayashi, T. Kudo, S. Koshimizu, M. Takahara, M. Iwami, Y. Watanabe, H. Sekimoto, K. Yano et al. (2019) Expression of genes from paternal alleles in rice zygotes and involvement of OsASGR-BBML1 in initiation of zygotic development. Plant Cell Physiol 60: 725–737. 10.1093/pcp/pcz030 30801122

[B59] Rattanawong, K., N. Koiso, E. Toda, A. Kinoshita, M. Tanaka, H. Tsuji and T. Okamoto (2021) Regulatory functions of ROS dynamics via glutathione metabolism and glutathione peroxidase activity in developing rice zygote. Plant J 108: 1097–1115. 10.1111/tpj.15497 PMC9293154 34538012

[B60] Rattanawong, K., K. Totsuka, A. Satoh, S. Koshimizu, K. Yano and T. Okamoto (2026) Autonomous development and regeneration of isolated rice egg cells in a fertilization-independent manner. J Exp Bot 77: 463–481. 10.1093/jxb/eraf406 PMC12794267 40972645

[B61] Russell, S.D. (1992) Double fertilization. Int Rev Cytol 40: 357–390.

[B62] Sambatti, J.B., D. Ortiz‐Barrientos, E.J. Baack and L.H. Rieseberg (2008) Ecological selection maintains cytonuclear incompatibilities in hybridizing sunflowers. Ecol Lett 11: 1082–1091. 10.1111/j.1461-0248.2008.01224.x PMC2737365 18643842

[B63] Sato, A., K. Toyooka and T. Okamoto (2010) Asymmetric cell division of rice zygotes located in embryo sac and produced by in vitro fertilization. Sex Plant Reprod 23: 211–217. 10.1007/s00497-009-0129-9 20054584

[B64] Scholten, S., H. Lörz and E. Kranz (2002) Paternal mRNA and protein synthesis coincides with male chromatin decondensation in maize zygotes. Plant J 32: 221–231. 10.1046/j.1365-313x.2002.01418.x 12383087

[B65] Seguí-Simarro, J.M., N.M.A. Jacquier and T. Widiez (2021) Overview of in vitro and in vivo doubled haploid technologies. Methods Mol Biol 2287: 3–22. 10.1007/978-1-0716-1315-3_1 34270023

[B66] Sugiura, R., H. Tarutani, A. Satoh, T. Maryenti, T. Okamoto and T. Ishii (2025) Identification of mitochondrial genome factors controlling the phenotype of rice-wheat cybrid plant. Breed Res 27 (Suppl 2): 79 (in Japanese).

[B67] Sukawa, Y. and T. Okamoto (2018) Cell cycle in egg cell and its progression during zygotic development in rice. Plant Reprod 31: 107–116. 10.1007/s00497-017-0318-x 29270910

[B68] Sun, M., H. Yang, C. Zhou and H.U. Koop (1995) Single-pair fusion of various combinations between female gametoplasts and other protoplasts in *Nicotiana tabacum*. Acta Bot Sin 37: 1–6 (in Chinese with English summary).

[B69] Sun, M.X., A. Moscatelli, H.Y. Yang and M. Cresti (2000) In vitro double fertilization in *Nicotiana tabacum* (L.): polygamy compared with selected single pair somatic protoplast and chloroplast fusions. Sex Plant Reprod 13: 113–117.

[B70] Tian, H.Q. and S.D. Russell (1997) Micromanipulation of male and female gametes of *Nicotiana tabacum*: II. Preliminary attempts for in vitro fertilization and egg cell culture. Plant Cell Rep 16: 657–661. 10.1007/BF01275510 30727614

[B71] Toda, E., Y. Ohnishi and T. Okamoto (2016) Development of polyspermic rice zygotes. Plant Physiol 171: 206–214. 10.1104/pp.15.01953 PMC4854695 26945052

[B72] Toda, E., Y. Ohnishi and T. Okamoto (2018) Effects of an imbalanced parental genome ratio on development of rice zygotes. J Exp Bot 69: 2609–2619. 10.1093/jxb/ery094 PMC5920335 29538694

[B73] Toda, E., N. Koiso, A. Takebayashi, M. Ichikawa, T. Kiba, T. Osakabe, Y. Osakabe, H. Sakakibara, N. Kato and T. Okamoto (2019) An efficient DNA- and selectable marker-free genome editing system using zygotes in rice. Nat Plants 5: 363–368. 10.1038/s41477-019-0386-z 30911123

[B74] Toda, E., S. Koshimizu, A. Kinoshita, T. Higashiyama, T. Izawa, K. Yano and T. Okamoto (2025) Transcriptional dynamics during karyogamy in rice zygotes. Development 152: DEV204497. 10.1242/dev.204497 PMC11829756 39777484

[B75] Tsunewaki, K. (2009) Plasmon analysis in the Triticum–Aegilops complex. Breed Sci 59: 455–470.

[B76] Tsunewaki, K., G.Z. Wang and Y. Matsuoka (2002) Plasmon analysis of Triticum (wheat) and Aegilops. 2. Characterization and classification of 47 plasmons based on their effects on common wheat phenotype. Genes Genet Syst 77: 409–427. 10.1266/ggs.77.409 12589076

[B77] Uchiumi, T., I. Uemura and T. Okamoto (2007) Establishment of an in vitro fertilization system in rice (*Oryza sativa* L.). Planta 226: 581–589. 10.1007/s00425-007-0506-2 17361458

[B78] Wang, Y.Y., A. Kuang, S.D. Russell and H.Q. Tian (2006) In vitro fertilization as a tool for investigating sexual reproduction of angiosperm. Sex Plant Reprod 19: 103–115.

[B79] Yamada, H., N. Kato, M. Ichikawa, K. Mannen, T. Kiba, Y. Osakabe, H. Sakakibara, M. Matsui and T. Okamoto (2024) DNA- and selectable-marker-free genome-editing system using zygotes from recalcitrant maize inbred B73. Plant Cell Physiol 65: 729–736. 10.1093/pcp/pcae010 38288629

